# Structure and function of the topsoil microbiome in Chinese terrestrial ecosystems

**DOI:** 10.3389/fmicb.2025.1595810

**Published:** 2025-08-25

**Authors:** Yuqiang Li, Yulong Duan, Junbiao Zhang, Evangelos Petropoulos, Jianhua Zhao, Fasi Wu, Lilong Wang, Yun Chen, Xuyang Wang

**Affiliations:** ^1^State Key Laboratory of Ecological Safety and Sustainable Development in Arid Lands, Northwest Institute of Eco-Environment and Resources, Chinese Academy of Sciences, Lanzhou, China; ^2^Naiman Desertification Research Station, Northwest Institute of Eco-Environment and Resources, Chinese Academy of Sciences, Tongliao, China; ^3^University of Chinese Academy of Sciences, Beijing, China; ^4^Shanghai Majorbio Bio-Pharm Technology Co., Ltd., Shanghai, China; ^5^Stantec, Ltd., Newcastle upon Tyne, United Kingdom; ^6^National Research Center for Conservation of Ancient Wall Paintings and Earthen Sites, Dunhuang Academy, Dunhuang, China

**Keywords:** terrestrial ecosystem, metagenomics, functional genes, SOC decomposition and biosynthesis, N cycle, S cycle

## Abstract

While soil microorganisms underpin terrestrial ecosystem functioning, how their functional potential adapts across environmental gradients remains poorly understood, particularly for ubiquitous taxa. Employing a comprehensive metagenomic approach across China’s six major terrestrial ecosystems (41 topsoil samples, 0–20 cm depth), we reveal a counterintuitive pattern: oligotrophic environments (deserts, karst) harbor microbiomes with significantly greater metabolic pathway diversity (KEGG) compared to resource-rich ecosystems. We provide a systematic catalog of key functional genes governing biogeochemical cycles in these soils, identifying: 6 core CAZyme genes essential for soil organic carbon (SOC) decomposition and biosynthesis; 62 nitrogen (N)-cycling genes (KOs) across seven critical enzymatic clusters; 15 sulfur (S)-cycling genes (KOs) within three key enzymatic clusters. These functional gene abundances exhibit distinct, geography-driven clustering patterns, strongly correlated with eight environmental drivers (latitude, NDVI, pH, EC, SOC, TN, C:N ratio, and MAP). This work provides a predictive framework and actionable genetic targets (e.g., specific CAZyme, N/S cycling genes) for potentially manipulating soil microbiomes to enhance ecosystem resilience and biogeochemical functions under stress.

## 1 Introduction

Terrestrial ecosystems ensure satisfactory quality on air, water and nutrition for humans as well as other organisms to thrive (Hu et al., 2024). Biogeochemical cycling of elements such as carbon (C), nitrogen (N), and sulfur (S) influence the dynamic equilibrium and availability of compounds’ turnover in the terrestrial biosphere. Soil is the largest pool of organic matter on earth, storing ~1,500 giga-tonnes (Gt) C, mass equal to the carbon present in the atmosphere (~750 Gt C) and as vegetation (~560 Gt C) combined (Crowther et al., 2019). Minor changes in the global soil C pool results to significant changes in carbon dioxide (CO_2_) concentration in the atmosphere affecting C-cycle, and thus, contributing to climate change (Luo et al., 2019). Soil C pool is conceptually divided into two fractions: (i) soil organic carbon (SOC) and (ii) soil inorganic carbon (SIC), with the former considered more active than the latter. Nitrogen (N) is essential for all living organisms, crucial for the biosynthesis of key cellular components such as proteins and nucleic acids (Kuypers et al., 2018; Nelson et al., 2016). The role of sulfur (S) and N in cell nutrition appears very similar. Both elements are utilized by living organisms (i.e., plants and plant-like microorganisms) to form proteins and other macromolecules (Evans, 2012; Schiff and Fankhauser, 1981; Wallace and Edmonds, 2011). As two of the main limiting nutrients in terrestrial ecosystems, N and S affect microbial mineralization of SOC by directly regulating the growth of plants and microorganisms altering their structure and function (Gao et al., 2024; Kopittke et al., 2017; Xu et al., 2021). Generally, C, N, and S cycling in terrestrial ecosystems could be affected remarkably by multiple global change factors (GCFs), such as N deposition, temperature, and precipitation regimes (frequency/intensity) (Chen et al., 2016; García et al., 2013; Grau-Andrés et al., 2021; Wu et al., 2022; Yue et al., 2017; Zeglin et al., 2013). Nevertheless, the impact of multiple GCFs on soil C, N, and S cycling, particularly between distinct ecological regions, is not yet fully understood. This is largely due to the non-standardized approaches in research methods and experimental scales. Accordingly, one of the most fundamental, yet challenging, issues is to describe and predict the cycling processes of C, N, and S (as well as other elements) across the world’s terrestrial ecosystems.

Microorganisms predominate in all natural environments playing vital roles in all biogeochemical cycles due to their diverse speciation, their wide distribution, and different metabolic patterns (Santos-Júnior et al., 2020; Sokol et al., 2022). For microbial ecologists, however, it remains a significant challenge to establish detailed connections between the soil microbiome and the processes of SOC decomposition and biosynthesis, as well as with both the N and S cycles. For the last two decades, investigation of biogeochemical element cycling *vis-à-vis* microbial activity mainly relied on sequencing of functional genes and Geochip technologies. These techniques, however, rely on primers and probes, respectively, which have inherent limitations such as specificity-related biases and low coverage. These issues make it challenging to comprehensively interpret the dynamics of SOC, N, and S. Recent advances in metagenomic techniques though, provide with unprecedented opportunities to investigate microbial functional compositions in depth, utilizing short-read next-generation sequencing data, benefiting from the ability to rapidly quantify thousands of notably transmissible resistances genes in a single sample (Hendriksen et al., 2019). Likewise, the new technological breakthroughs can provide additional information regarding the presence of soil microbial species, pathogens, and virulence genes, whose data can be readily analyzed should novel genes of interest be identified. Admittedly however, short reads metagenomics provide limited information regarding the genes’ host or the genetic environment. Previously, metagenomic techniques were used to reveal the microbial communities involved in the cycling of C (as SOC), N and S in multiple ecosystems (Anantharaman et al., 2018; Dai et al., 2021; Murakami et al., 2022; Santos-Júnior et al., 2020; Song et al., 2022), including river, mountains, and oceans. Metagenomic techniques are mostly used to reveal the characteristics of soil C, N, and S cycles in engineered ecosystems (i.e., farmlands) or other similar environments heavily affected by anthropogenic activity (Bender et al., 2023; Hu et al., 2022; Liu J. J. et al., 2023; Su et al., 2017). Thus far, little is known about the characteristics of soil C, N, and S cycles and their geographical distribution patterns in ecosystems less disturbed by human activity, such as in deserts, forests, and grasslands (Coleine et al., 2024; Cui et al., 2024; Liu H. Y. et al., 2023). Similar studies on soil C, N, and S cycling in more conventional/engineered ecosystems assisted in comprehensive understanding of the microbial functions; this highlighted the usefulness of the approach—therefore, it is key to repeat such type of studies on those more “virgin” ecosystems.

China is one of the richest countries in the world in terms of terrestrial ecosystem types with a wide variety of geographical terrains. For example, the Qinghai–Tibet Plateau (Ji et al., 2020), Loess Plateau (Yang et al., 2022; Zhong et al., 2022), and Hexi Corridor (Jiao et al., 2022), all of which are typical eco-regions, each constituting an ecotone. In general, an ecotone refers to the transition area at the interface of two rather different ecosystems; an ecotone has poor stability, weak ability to resist or recover from disturbance, and is prone to degradation. In China, ecotone areas are mainly split to (i) the agro-pasture ecotone, (ii) the forest-grass ecotone, and the (iii) agro-forestry ecotone (Liu et al., 2015; Sun et al., 2019; Wang et al., 2019). In a recent study, we employed next-generation sequencing (Illumina MiSeq PE300 platform) (Ren et al., 2022) to investigate the biogeographic patterns of topsoil (0–20 cm) microbiomes across six distinct Chinese eco-regions, based on an extensive field survey. The research encompassed taxonomic characterization and examined the divergent drivers of β-diversity in both bacterial and eukaryotic communities (Duan et al., 2025). Yet, understanding of the variety and prevalence of functional traits associated with soil microbiomes across China’s typical eco-regions, including type, abundance, spatial patterns, functional genes and key environmental drivers, is still lacking. To fill that knowledge gap, the present study aims to address these uncertainties by performing metagenomics in a large-scale soil survey. Due to great environmental heterogeneity, we tried to test the hypothesis whether, the six distinct Chinese eco-regions would result in different soil microbial genes and soil organic carbon (SOC), nitrogen (N), and sulfur (S) metabolic pathways.

## 2 Materials and methods

### 2.1 Study area and soil sampling

For the hereby work, six representative eco-regions of China were sampled for study, specifically: (i) the karst area of southwestern China (KS), (ii) the agro-pastoral ecotone of southwestern China (AS), (iii) the Qinghai–Tibet Plateau (QT), (iv) the Loess Plateau (LP), the (v) forest-grassland ecotone (FG), and the (vi) deserts of Hexi Corridors (HC) (Supplementary Figure S1). The selected areas have a mean annual temperature spanning from −22.9°C to 28.6°C, a mean annual precipitation of 44.9–1815 mm, and an elevation that ranges from 27 m to 8,305 m a.s.l. The region exhibits distinct climatic and vegetation zones from east to west, shaped by climatic conditions and soil characteristics: subtropical monsoon climate (primarily encompassing KS and parts of AS) and plateau mountain climate (spanning parts of AS and QT) dominate eastern areas. KS and AS feature expansive yellow-brown, red, and cinnamon soils, supporting subtropical evergreen-deciduous broad-leaved forests and alpine meadows. QT’s black felty soils and chernozem foster alpine grasslands. Transitioning westward, temperate continental monsoon climate (LP, FG, HC) emerges. LP’s brown calcic, aeolian sandy, and loessal soils sustain temperate grasslands, sandy semi-shrub grasslands, and warm temperate deciduous broad-leaved forests. FG’s boggy/meadow soils and dark-brown/aeolian sandy soils host similar warm temperate deciduous broad-leaved forests. The westernmost Calcic-Orthic Aridosols in HC create desert grassland ecosystems (Duan et al., 2025).

Soil sampling took place from June to July 2019 and during July 2020. Based on the mean annual precipitation gradient, a total of *n* = 41 locations (6 locations for KS, and likewise 6 for AS, 9 for QT, 5 for LP, 6 for FG, and 9 for HC) were chosen at intervals of approximately 100–110 km along four transects (Supplementary Figure S1). All samples collected from the minimally disturbed natural soils were collected during the peak of the growing season. At each location, surface litter was removed within a 10 × 10 m plot, then along the plot’s diagonal line five 1 × 1 m quadrats were established. From each quadrat, along that diagonal line, three replicate soil samples (0–20 cm depth) were obtained and homogenized to provide a single composite soil sample per plot. All composite soil samples were individually packed in sterilized polyethylene bags, and taken rapidly to the lab using portable refrigerators. Each composite soil sample was then split into two subsamples: one that was stored at 4°C for later biochemical analysis, the other was stored at −80°C prior DNA extraction and molecular analysis. All relevant variables and location information of our study’s soil samples are detailed in Supplementary Table S1.

### 2.2 Data collection—climatic factors and soil physicochemical properties

The MAP (mean annual precipitation), MAT (mean annual temperature), and NDVI (normalized difference vegetation index) data for the wider experimental area were obtained from the Chinese Meteorological Database.^1^ Soil pH was measured using an E20-FiveEasy pH meter (Mettler Toledo, Giessen, Germany). EC (electrical conductivity), an indicator of the soluble salt content of soil, was measured using an electric conductometer. Both soil parameters were measured using a soil-water suspension (5:1, v/v mixture of deionized water and fresh soil) after the samples were subjected to shaking for 30 min. Both the content of SOC (soil organic carbon) and TN (soil total nitrogen) were quantified using a carbon-hydrogen-nitrogen elemental analyzer (2400 II CHN Elemental Analyzer, Perkin Elmer, Boston, MA, United States). Climatic factors and soil properties datasets are summarized in Supplementary Table S1.

### 2.3 Molecular analysis—DNA extraction, library construction, and metagenomic sequencing

Total genomic DNA was extracted from each soil sample using the E.Z.N.A.^®^ Soil DNA Kit (Omega Bio-Tek, Norcross, GA, United States) following the manufacturer’s instructions. The concentration and purity of the extracted DNA was determined using TBS-380 and NanoDrop2000 spectrophotometers. DNA extract quality was checked on 1% agarose gel.

The DNA extracts were fragmented to an average size of about 400 bp, using the Covaris M220 (Gene Company Limited, China). For the paired-end library construction, NEXTFLEX Rapid DNA-Seq (Bio Scientific, Austin, TX, United States) was used. Adapters containing the full complement of sequencing primer hybridization sites were ligated to the blunt-end of each fragment. Next, the paired-end sequencing was carried out on an Illumina HiSeq Xten system (Illumina Inc., San Diego, CA, United States) at the Majorbio Bio-Pharm Technology Co., Ltd. (Shanghai, China) by using HiSeq X Reagent Kits and following the manufacturer’s instructions.^2^

### 2.4 Sequencing data processing, assembly, and annotation

The generated data from sequencing were analyzed on the free Majorbio Cloud Platform online^3^ (Ren et al., 2022). Briefly, the paired-end Illumina reads were trimmed of their adaptors, and any low-quality reads (i.e., having a length <50 bp, or a quality value <20, or N bases) were removed by the fastp (v 0.20.0) tool^4^ (Chen et al., 2018). The resulting high-quality read pairs from the same sample were then assembled into contigs by using MEGAHIT (v1.1.2) software^5^ (Li et al., 2015) with kmer values ranging from 47 to 97 (step = 10). Contigs having a length ≥300 bp were selected for gene prediction and functional annotation.

An open reading frame (ORF) for each contig was predicted by MetaGene^6^ (Noguchi et al., 2006). Those predicted ORFs with a length ≥100 bp were retrieved and translated into amino acid sequences using the NCBI translation table. Next, a non-redundant gene catalog was constructed using CD-HIT (v4.6.1)^7^ (Fu et al., 2012) based on a minimal 90% sequence identity and 90% coverage. To calculate the abundance of genes at a 95% identity threshold, the obtained high-quality reads were aligned to non-redundant gene catalogs via a SOAP aligner (v 2.21)^8^ (Li et al., 2008). For their taxonomic identification, Diamond (v0.8.35)^9^ (Buchfink et al., 2015) was used with an *e*-value <1 × 10^−5^ and the alignments searched against the NCBI microbial NR database. The predicted gene fragments were searched against KEGG,^10^ NCycDB (Tu et al., 2019) and SCycDB (Yu et al., 2021) reference databases using Diamond (*e*-value <1 × 10^−5^) for functional annotation. Hmmscan^11^ was used to search against the Carbohydrate-Active Enzymes (CAZy) database^12^ (*e*-value cutoff = <1 × 10^−5^).

To minimize the effects of sequencing depth on statistical analysis, the calculation method of species and gene abundance is Reads Per Kilobase Million (RPKM) (Ye et al., 2024):


$${\text{RPKM}}_{i}=\frac{R_{i}\times 10^{6}}{L_{i}\times \sum_{1}^{n}R_{j}}$$

where *R_i_* represents the abundance value of Gene*_i_* in a given sample, i.e., the number of Reads compared to Gene*_i_* in that sample; *L_i_* means the nucleotide length of Gene*_i_*; and $\sum_{1}^{n}R_{j}$ represents the sum of reads corresponding to all genes in that sample.

### 2.5 Statistical analysis

Microsoft Excel 2019 and *R* (v 3.2.1) were used for statistical data analyses. The differences in Chao1 index for the α-diversity of taxonomic (species) and functional traits [KOs (KEGG Orthology) and CAZyme-encoding genes] across the six eco-regions were performed by using the nonparametric Kruskal–Wallis test and the post-hoc pairwise Wilcoxon rank-sum test, with *p*-values adjusted through the Benjamini–Hochberg method. The α-diversity^13^ estimates were calculated using the diversity function of the “vegan” package^14^ in the *R* computing platform (v 3.2.1)^15^ (Oksanen, 2017). Kruskal–Wallis test and Wilcoxon rank-sum test were performed through functions “kruskal.test” and “wilcox.test” in package “stats” in *R* (v 3.2.1). To identify taxa and metabolic pathways as biomarkers, the linear discriminant analysis (LDA) effect size (LEfSe) algorithm (LDA >2.5, *p* < 0.05) (Segata et al., 2011) was applied. The abundance (i.e., RPKM, reads per kilobase per million mapped reads) of all microbial groups (bacteria, archaea, viruses, and eukaryota) at phylum level was visualized in histogram plot (s). RPKM abundances of differentially abundant (DA) KEGG pathways, normalized by *z*-score across all data sets, visualized using heatmap, and the sample-based dendrogram was performed using Euclidean distance metric in package “pheatmap”^16^ in R (v 3.2.1). Principal co-ordinates analysis (PCoA) of taxonomic traits (species) and functional traits (KOs, KEGG Orthology database), based on their Bray–Curtis (BC) distances, was carried out using the “vegan” package (see text footnote 14) in *R* (v 3.2.1) (Oksanen, 2017). The differences of taxonomic traits (species) and functional traits among six eco-regions were tested using permutational multivariate analysis of variance (PERMANOVA) with 9,999 permutations. Distance-based redundancy analysis (db-RDA) was implemented to explore the effects of environmental factors on the SOC’s decomposition and biosynthesis, N cycling, and S cycling across six eco-regions using the Bray–Curtis dissimilarities distance matrix. The individual effects of each explanatory variable on response variables from db-RDA were estimated using the hierarchical partitioning method (“rdacca.hp” function from rdacca.hp. package) (Lai et al., 2022).

## 3 Results

### 3.1 Microbiome genes from the topsoil of Chinese eco-regions

**Figure 1:**
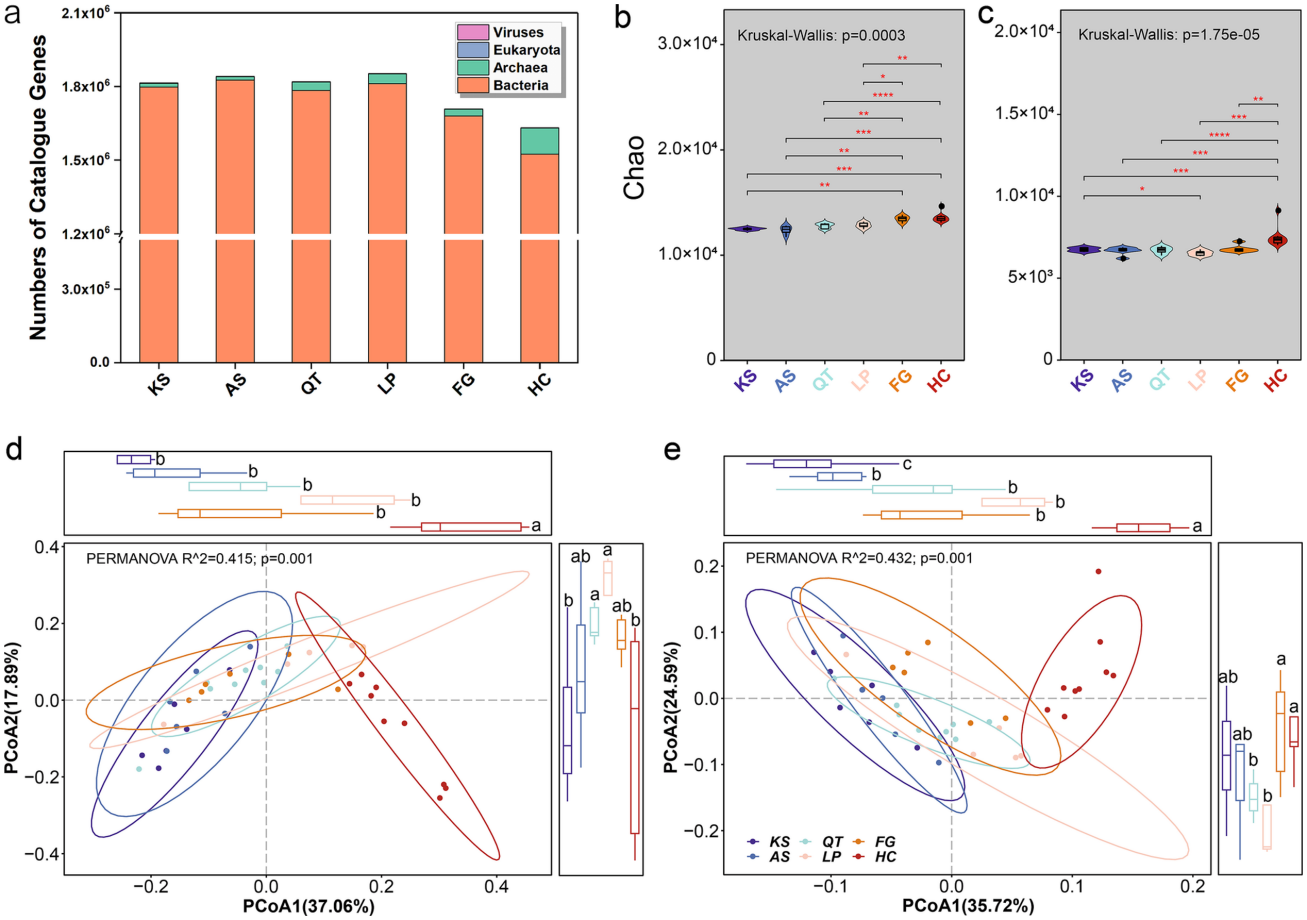
Overview of taxonomic and functional traits of soil microorganisms in six eco-regions across China. **(a)** Taxonomic classification of the 10,669,587 non-redundant genes across all regions. **(b,c)** Show the Chao1 index for the α-diversity of taxonomic (species) and functional traits (KOs, KEGG Orthology) for each region, respectively (the differences in Chao1 index across the six eco-regions were performed by using the nonparametric Kruskal–Wallis test and the post-hoc pairwise Wilcoxon rank-sum test, with *p*-values adjusted through the Benjamini–Hochberg method; ^*^p < 0.05; ^**^0.01 < *p* < 0.05; ^***^0.001 < *p* < 0.01; ^****^p < 0.001). The PCoA based on Bray–Curtis distances was plotted to display the β-diversity of taxonomic traits (species) **(d)** and functional traits (KOs, KEGG Orthology) **(e)** across all 41 sampling sites. The *R*^2^ and *p*-values were calculated using PERMANOVA (9,999 permutations) and are indicated in each plot. The differences in PCoA1/PCoA2 across the six eco-regions were performed by using the nonparametric Kruskal–Wallis test and the post-hoc pairwise Wilcoxon rank-sum test, with *p*-values adjusted through the Benjamini–Hochberg method. KS, karst area of southwest China; AS, agro-pastoral ecotone of southwest China; QT, Qinghai–Tibet Plateau; LP, Loess Plateau; FG, forest-grassland ecotone; HC, deserts of the Hexi Corridor.

Topsoil samples were collected from *n* = 41 locations, covering six distinct geographical regions (Supplementary Figure S1 and Supplementary Table S1). 579.3 gigabases (Gb) of paired-end sequence data were generated averaging 93.6 million paired reads per sample. *De novo* assembly of sequencing data yielded a non-redundant gene catalog for all 41 locations. The total length of this non-redundant assembly was 24.6 Gb (means contig N50 length of 543 bp), from which 54.7 million partial genes >100 bp were predicted. After removing redundancy by clustering the genes by identity (>90%), and by shortening gene coverage (>90%), a total of 25.4 million non-redundant genes were deposited in the metagenomic libraries. Bacterial genes were the most predominant among all species comprising 97.69% of all sequences, followed by 2.22% for archaea, 0.07% for eukaryota, and only 0.02% for viruses (Figure 1a and Supplementary Table S2). Our results showed that the number of genes sequenced had their lowest abundance in the Hexi Corridor deserts (region HC) (1632676). Regarding the five other eco-regions, gene abundance ranged as FG, LP, QT, AS, and KS from lowest to highest, respectively. The low abundance at the desert ecosystem could be attributed to the harsh conditions present, characterized by extreme drought, extreme temperature variation (e.g., great thermal difference between day and night), and low soil fertility (Coleine et al., 2024; D’Odorico et al., 2013; Duan et al., 2022).

The Chao1 index values for taxonomic (species) and functional (KOs, KEGG Orthology) traits for all microbial groups from the trialed eco-regions are shown in Figures 1b,c respectively. For taxonomic traits, the Chao1 richness of the entire soil microbiome ranged from 12383.95 (at AS group) to 13569.53 (at HC group) with an overall mean (±SD) and median of 12961.03 ± 551.81 and 12938.09, respectively (Supplementary Tables S3, S5). For functional traits, whole microbiome’s Chao1 richness based on the KOs ranged from 6540.04 (at LP group) to 7505.37 (at HC group) having an overall mean of 6875.65 ± 476.89 and a median of 6771.20 across the six regions (Supplementary Tables S4, S6). Significant differences on soil microbiome’s Chao1 indices (species or KOs) between the selected region samples (Supplementary Tables S5, S6). Overall, the Chao1 index for the α-diversity of taxonomic and functional traits at HC and FG was higher than that from other regions. Interestingly, although soil microbial abundance and diversity in desert ecosystems are generally lower than that in less stressful environments, Chao1 richness of taxonomic traits (species) and their functional traits (KOs) in the HC region exceeded that from the other five regions (KS, AS, QT, LP, and FG) (Figures 1b,c).

Principal coordinates analysis (PCoA) revealed significant differences at the β-diversity of both taxonomic (species) (PERMANOVA, *R*^2^ = 0.415, *p* = 0.001; Figure 1d) and functional traits (KOs) (PERMANOVA, *R*^2^ = 0.432, *p* = 0.001; Figure 1e) between different regions. Evidently, whether taxonomic or functional, the microbiome traits from the five regions (KS, AS, QT, LP, and FG) were geographically separated along the first principal coordinate from those traits of HC. Overall, the β-diversity of taxonomic and functional traits at HC was obviously different from other regions (Figures 1d,e). Again, these pronounced differences are mainly attributed to the unique conditions of the desert habitat (D’Odorico et al., 2013; Hu et al., 2019).

**Figure 2:**
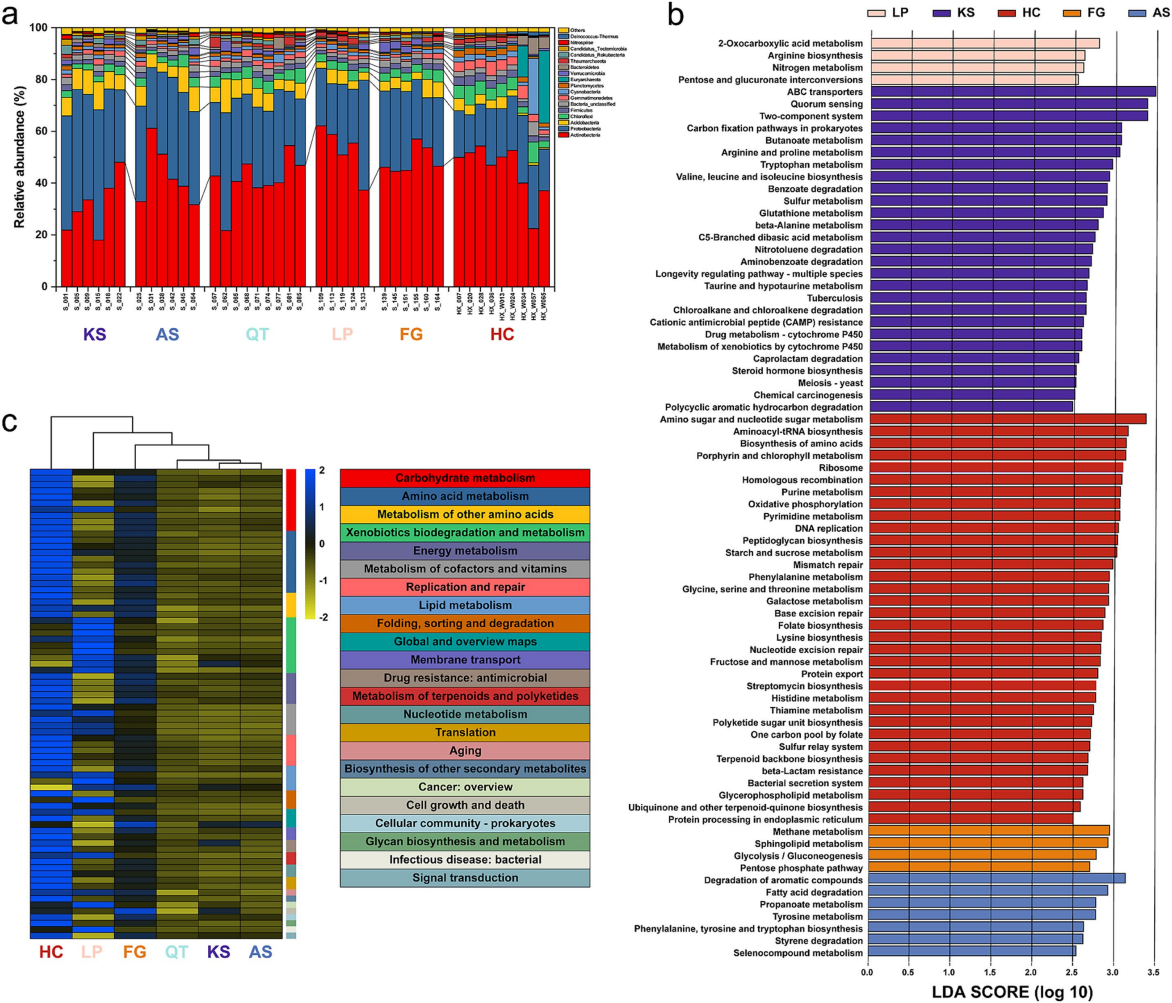
Taxonomic composition and functional gene potential of the 41 sampling sites in six eco-regions across China. **(a)** Relative abundance of the major taxonomic groups at the phylum level. Cases of a relative abundance, <1% were subsumed into “others.” **(b)** KEGG pathways significantly enriched in the six regions using the linear discriminant analysis (LDA) effect size (LEfSe) method (LDA >2.5, *p* < 0.05). **(c)** RPKM abundances of differentially abundant (DA) KEGG pathways, normalized by *z*-score across all data sets. UPGMA clustering of groups (top) was based on Pearson correlations. KS: karst area of southwest China. AS, agro-pastoral ecotone of southwest China; QT, Qinghai–Tibet Plateau; LP, Loess Plateau; FG, forest-grassland ecotone; HC, deserts of the Hexi Corridor.

Taxonomic analysis identified a total of 105 phyla of soil microorganisms, specifically: 82 for bacteria, 12 for archaea, 10 for eukaryota, plus 1 virus (Figure 2a and Supplementary Table S7). Most of the metagenomes were dominated by *Actinobacteria* (18.01–62.09%) and *Proteobacteria* (14.66–50.31%), followed by *Acidobacteria* (0.61–13.10%). In addition, *Chloroflexi* (1.37–8.01%), *Firmicutes* (1.19–3.78%), Bacteria_unclassified (0.96–5.42%), *Gemmatimonadetes* (0.65–5.49%), *Cyanobacteria* (0.89–21.54%), *Planctomycetes* (0.72–2.54%), *Euryarchaeota* (0.19–27.94%), *Verrucomicrobia* (0.23–3.85%), *Bacteroidetes* (0.47–6.38%), *Thaumarchaeota* (0.05–3.90%), *Candidatus_Rokubacteria* (0.05–3.45%), *Candidatus_Tectomicrobia* (0.14–2.36%), *Nitrospirae* (0.15–1.70%), and *Deinococcus-Thermus* (0.22–1.51%), together accounted for ca. 98% of all metagenomic sequences derived from the topsoil samples (Supplementary Table S7). Overall, these bacterial phyla exhibit no site-specificity across China and are considered common in terrestrial ecosystems on a global scale (Delgado-Baquerizo et al., 2018).

The RPKM abundance data were screened for the KEGG pathways enriched in the six eco-regions, observation based on the linear discriminant analysis (LDA) effect size (LEfSe) method (LDA >2.5, *p* < 0.05). Collectively, 76 KEGG pathways (18.5% of total) were enriched in the six regions, with 4, 27, 34, 4, 7, and 0 pathways enriched in LP, KS, HC, FG, AS, and QT regions, respectively (Figure 2b and Supplementary Table S8). These 76 KEGG pathways could be classified into 23 classes that are mainly associated with seven biological processes (Figure 2c and Supplementary Table S8): carbohydrate metabolism amino acid metabolism xenobiotics biodegradation and metabolism, energy metabolism, metabolism of cofactors and vitamins, replication and repair lipid metabolism (Figure 2c and Supplementary Table S8). The RPKM-normalized read counts notably differed (more than five-fold) across the six regions, being 7.29 × 10^6^, 6.27 × 10^6^, 1.22 × 10^7^, 8.63 × 10^6^, 6.44 × 10^6^, and 6.37 × 10^6^ in LP, KS, HC, FG, AS, and QT, respectively (Supplementary Table S8). Additionally, the hereby results reveal that different eco-regions may form different functional metabolic niches. The heatmap analysis reveals that the three KS, AS, and QT regions in southern China are clustered together; likewise, the HC, LP, and FG in northern China are clustered together (Figure 2c and Supplementary Table S8).

### 3.2 Profile of the functional genes associated with C, N, and S cycling

To identify the functional genes involved in C, N, and S cycling, the obtained soil metagenomic reads were annotated using the databases of CAZy (Cantarel et al., 2008), KEGG (Kanehisa et al., 2008), NCycDB (Tu et al., 2019) and SCycDB (Yu et al., 2021).

**Figure 3:**
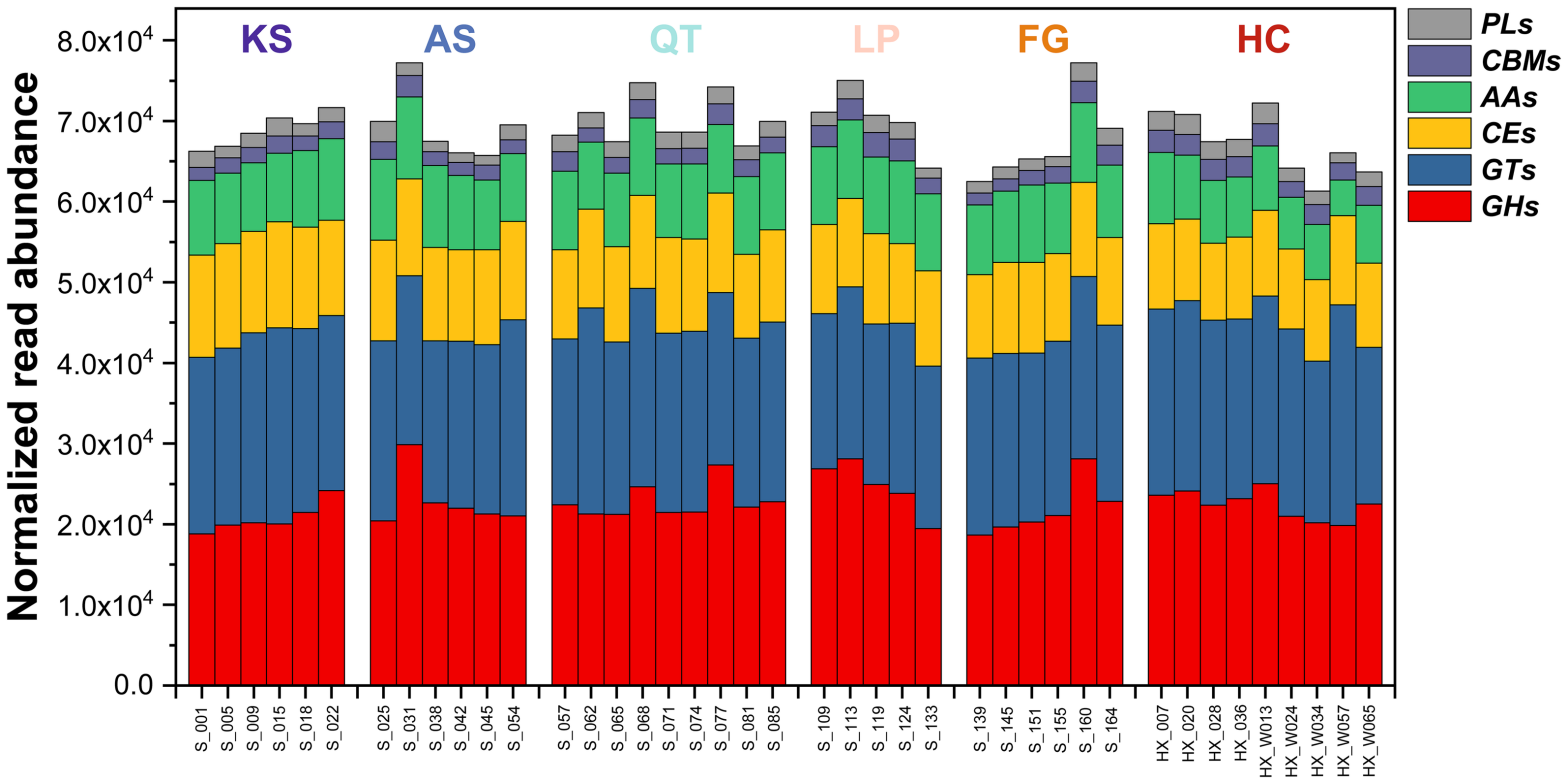
The RPKM abundance of functional trait genes relevant to SOC (soil organic carbon) decomposition and biosynthesis across the 41 sampled soil microbiomes in six eco-regions across China. GHs, glycoside hydrolases; GTs, glycosyltransferases; PLs, polysaccharide lyases; CEs, carbohydrate esterases; AAs, auxiliary activities; CBMs, carbohydrate-binding modules; KS, karst area of southwest China; AS, agro-pastoral ecotone of southwest China; QT, Qinghai–Tibet Plateau; LP, Loess Plateau; FG, forest-grassland ecotone; HC, deserts of the Hexi Corridor.

For carbohydrate metabolism, the 41 soil samples contained six critical CAZyme-encoding genes involved in SOC decomposition and biosynthesis (Figure 3; Supplementary Figure S2 and Supplementary Table S9). The genes had uneven distribution among the samples. Specifically, as gene and location in brackets: glycoside hydrolases (GHs), glycosyl transferases (GTs), polysaccharide lyases (PLs), carbohydrate esterases (CEs), auxiliary activities (AAs), and carbohydrate-binding modules (CBMs). This differentiation generally reflects the microbial substrate affinity, and as a proxy, the decomposition and biosynthesis potential of each community against SOC (Kanehisa and Goto, 2000). Evidently, the genes encoding the GHs (organic carbon decomposition) and GTs (organic carbon biosynthesis) enzymes were the most abundant in all samples across the six regions (Figure 3; Supplementary Figure S2), followed by those coding for CEs and AAs, and last but not least for CBMs and PLs. In addition, there has been considerable variation in the abundance of genes participating in SOC decomposition and biosynthesis across all 41 samples (ranging from 61267.39 to 77190.28) (Figure 3; Supplementary Figure S2 and Supplementary Table S9).

**Figure 4:**
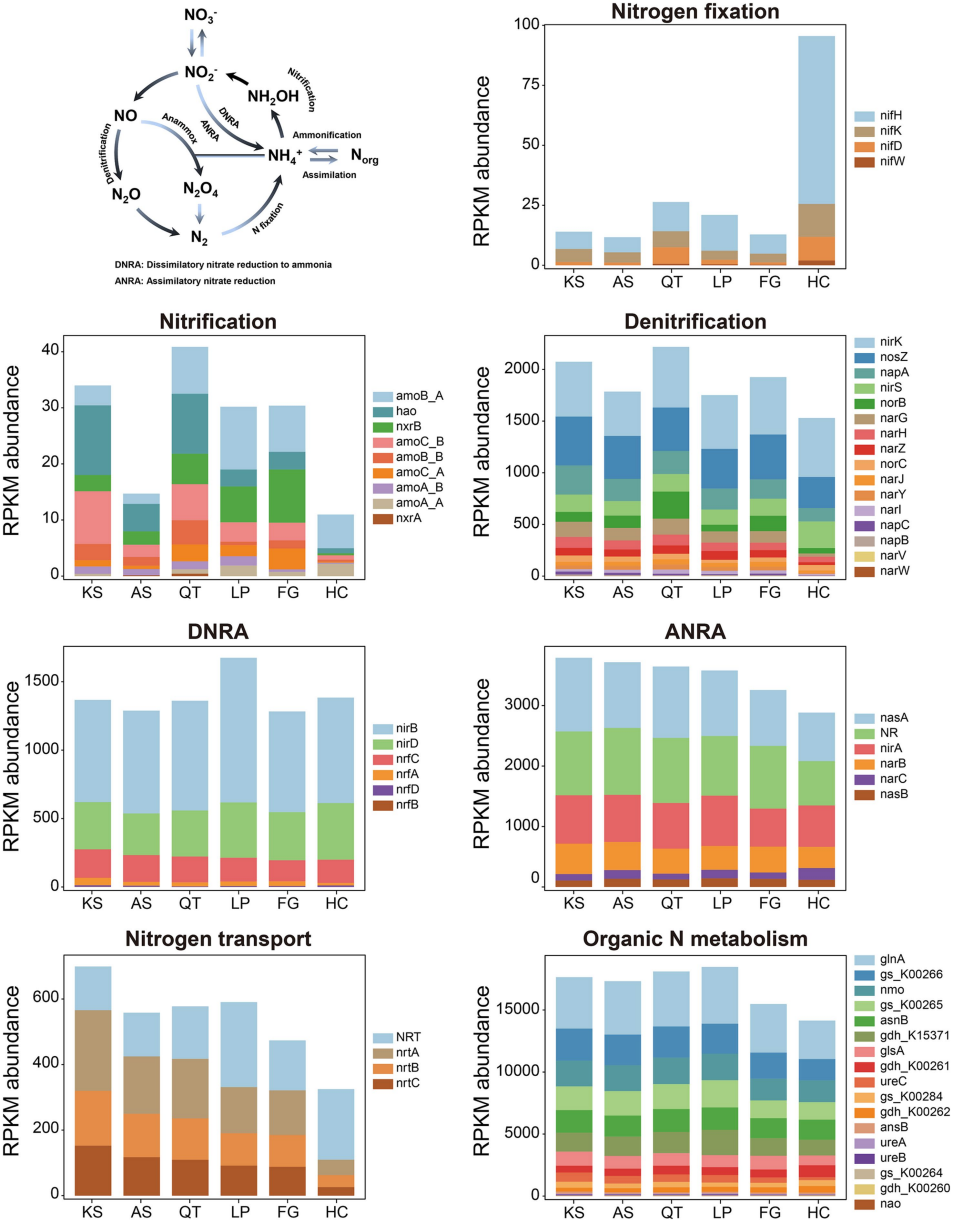
The N cycle and the RPKM abundance of functional trait genes related to N cycling in six eco-regions across China. DNRA, dissimilatory nitrate reduction to ammonia; ANRA, assimilatory nitrate reduction; KS, karst area of southwest China; AS, agro-pastoral ecotone of southwest China; QT, Qinghai–Tibet Plateau; LP, Loess Plateau; FG, forest-grassland ecotone; HC, deserts of the Hexi Corridor.

For nitrogen (N) metabolism, biogeochemical N cycling between inventories is often attributed to the six common N-transformation processes: *nitrogen fixation*, *nitrification*, *denitrification*, *nitrate reduction*, *nitrogen transport/nitrate assimilation*, and *organic nitrogen metabolism* (Kelly et al., 2021; Kuypers et al., 2018) (see the N-cycling model in Figure 4 and Table 1). Hereby, 62 gene families responsible for N cycling were detected from all samples across the six regions; these gene families could be categorized into 7 individual pathways (Figure 4 and Table 1; Supplementary Tables S10, S11). (1) *Nitrogen fixation*: 3 key gene families (*nifD*, *nifH*, and *nifK*) encoding the nitrogenase complex were identified in all regional groups (KS, AS, QT, LP, FG, and HC samples), *nifW* gene family was not present in either the KS or AS group. The highest and lowest abundance of gene families involved in nitrogen fixation were found in HC (95.52) and AS groups (11.68) respectively; the former nearly 9 times greater than the latter. Overall, the abundance of these four gene families was measurable yet consistently low across all 41 soil sites; (2) *Nitrification*: 7 key gene families (*amoB_A*, *amoB_B*, *amoC_A*, *amoC_B*, *nxrB*, and *hao*) encoding ammonia monooxygenase were identified in all six regions. *amoA_A* gene and *nxrA* gene families were identified in KS, AS, and QT. The gene families involved in nitrification were most abundant in the QT group (40.86), the least for HC (10.98) with the former 3.72 times higher than the latter. Among them, *amoB_A* and *hao* were the two most abundant gene families participating in the nitrification process; (3) *Denitrification*: 15 marker gene families (*nirK*, *nosZ*, *napA*, *nirS*, *norB*, *narG*, *narH*, *narZ*, *norC*, *narJ*, *narI*, *napC*, *napB*, and *narV*) encoding key enzymes for denitrification were identified in all six regions. One gene family *narW* was only identified in KS and AS. These gene families attained their highest abundance in the QT group (2217.52), being lowest in the HC (1529.79) with the former 1.45 times higher than the latter. The three most abundant gene families involved in the denitrification process were *nirK*, *nosZ*, and *napA*; (4) *Nitrate reduction*: 5 key functional gene families (*nirB*, *nirD*, *nrfC*, *nrfA*, and *nrfD*) encoding key enzymes for DNRA were identified in all six regions. One gene family *nrfB* was not present in HC. The highest abundance of these gene families involved in the DNRA pathway was found in the LP group (1674.78) and the lowest in the FG group (1283.15), with the former 1.07 times higher than the latter. Among them, *nirB* was the gene family present in greatest abundance. Meanwhile, 6 marker gene families (*nasA*, *NR*, *nirA*, *narB*, *narC*, and *nasB*) encoding key enzymes for ANRA pathway were detected in all six regions, with *nasA* and NR being the most abundant Furthermore, the highest abundance of gene families participating in the ANRA pathway occurred in the KS group (3789.56) and the lowest in FG (2883.24), the former was 1.31 times higher than the latter; (5) *Nitrogen transport*: 4 marker gene families (*NRT*, *nrtA*, *nrtB*, and *nrtC*) encoding key enzymes for nitrogen transport/nitrate assimilation were identified in all six regions. These gene families were found to be the most and least abundant in the KS (699.27) and HC (324.96) groups, respectively, the former being 2.15 times higher than the latter; (6) *Organic nitrogen metabolism*: 17 marker gene families (*glnA*, *gs_K00264*, *gs_K00265*, *gs_K00266*, *gs_K00284*, *nmo*, *asnB*, *gdh_K00260*, *gdh_K00261*, *gdh_K00262*, *gdh_K15371*, *glsA*, *ureA*, *ureB*, *ureC*, *ansB* and *nao*) encoding key enzymes for organic nitrogen metabolism were identified in all six regions. The highest abundance of these gene families was found in the LP group (18472.78) and the lowest in the HC group (14146.65); the former was 1.31 times higher than the latter. Of these, *glnA* and *gs_K00266* were the two most abundant genes involved in the organic nitrogen metabolism process. Thus, the significant differences in the total gene abundances of N metabolism among the different eco-regions were summarized, the list findings indicate that the soil microbiome of LP is the environment with the highest gene abundance for N metabolism (26120.01), followed by that of QT (25974.50), KS (25623.967), and AS (24699.21), with FG (22462.91) and HC (20375.07) having the lowest abundance (Figure 4 and Supplementary Table S10).

**Table 1:** Microbial nitrogen cycle processes in this study.

Name	Description	References
N fixation	The conversion of N_2_ to biologically available ammonia (NH_4_^+^) is carried out by the nitrogenase complex	Kelly et al. (2021) and Scott and Ludwig (2004)
Nitrification	Microbial enzymes (ammonia monooxygenase) catalyze the process whereby ammonia (NH_3_) is oxidized to nitrite (NO_2_^−^) and subsequently to nitrate (NO_3_^−^)	Casciotti et al. (2011), Tian et al. (2020), and van Kessel et al. (2015)
Denitrification	The conversion of NO_3_^−^ to N_2_ proceeds via four intermediate steps (NO_3_^−^ → NO_2_^−^ → NO → N_2_O → N_2_), producing several nitrogenous compounds with notable roles as air polluting gases (N_2_O and NO)	Skiba (2008), Tian et al. (2020), and van Kessel et al. (2015)
Nitrate reduction	The reduction of NO_3_^−^ to NH_4_^+^ ultimately leads to the incorporation of N into microbial biomass. Dissimilatory nitrate reduction to ammonia (DNRA) is an anaerobic process in which NO_3_^−^ serves as an electron acceptor to oxidize and release energy from organic carbon. It is mediated by nitrate reductases that form NO_2_^−^ and nitrite reductases that convert NO_2_^−^ to NH_4_^+^. DNRA is a novel biological pathway of N-cycling, and the shortest, in terrestrial ecosystems where NO_3_^−^ is reduced to NH_4_^+^ in soils. Compared with the DNRA, ANRA (assimilatory nitrate reduction) pathway is an energetically costly process that depends on different families of nitrate and nitrite reductases	Friedl et al. (2018), Kelly et al. (2021), and Pandey et al. (2020)
N transport	The *nrtABCD* gene cluster encodes an ATP-binding cassette (ABC)-type transporter capable of importing NO_3_^−^ or NO_2_^−^ from the extracellular environment	Kelly et al. (2021)
Organic N metabolism	Conversion of NH_4_^+^ to glutamate, glutamine, and urea	Galloway et al. (2008) and Kelly et al. (2021)

**Figure 5:**
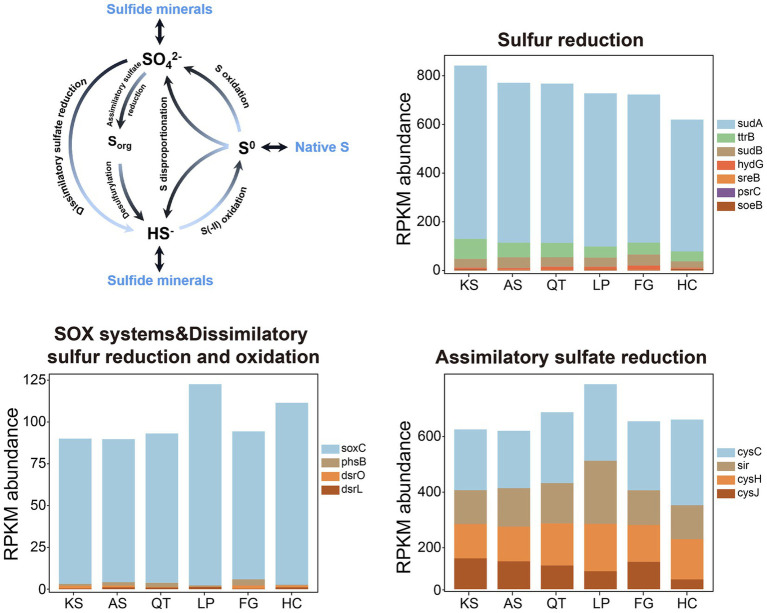
The S cycle and the RPKM abundance of functional trait genes related to S cycling in the six eco-regions across China. KS, karst area of southwest China; AS, agro-pastoral ecotone of southwest China; QT, Qinghai–Tibet Plateau, LP, Loess Plateau; FG, forest-grassland ecotone; HC, deserts of the Hexi Corridor.

For sulfur (S) metabolism, biogeochemical cycling of S between inventories is often attributed to three distinct sulfate-transforming processes (Figure 5 and Table 2): *sulfur assimilation*, *anaerobic sulfate respiration*, and *sulfide oxidation* (Llorens-Marès et al., 2015) (see the S-cycling model in Figure 5). In this study, from all soil samples across the six regions, a total of 15 gene families responsible for S cycling were detected. These gene families could be categorized into six individual pathways according to SCycDB (Yu et al., 2021) (Figure 5 and Table 2; Supplementary Tables S12, S13). (1) *Sulfur reduction*: 5 marker genes (*sudA*, *ttrB*, *sudB*, *hydG*, and *sreB*) encoding key enzymes participating in the Sulfur reduction pathway were identified in all six eco-regions. One gene family *psrC* with low abundance was not present in QT and FG. The highest abundance of these genes was found in the KS group (837.24) and the lowest in the HC group (614.17). Of these, *sudA* was the most abundant gene family associated with the S reduction pathway, 6.07 times of the total abundance of other genes in this pathway. (2) *Sulfur oxidation*: 1 marker gene family (*soeB*) encoding for key enzymes related to sulfite oxidation was identified in all six regions. Overall, the abundance of the gene family was measurable yet consistently low across all 41 sites. (3) *Sulfur disproportionation*: 1 marker gene family (*phsB*) with low abundance across all 41 sites. (4) *SOX systems*: 1 marker gene family (*soxC*) encoding for key enzymes related to thiosulfate oxidation was identified in all six regions. The highest and lowest abundance of these was found in the LP (108.81) and AS group (85.55) group respectively, the former being 1.27 times higher than the latter. (5) *Dissimilatory sulfur reduction and oxidation*: 2 marker gene families (*dsrO* and *dsrL*) with low abundance across all 41 soil sites. (6) *Assimilatory sulfate reduction*: 4 marker gene families (*cysC*, *cysH*, *cysJ*, and *sir*) encoding for key enzymes participating in the sulfate reduction were identified in all six eco-regions. The highest abundance of these genes was found in the LP group (736.94) and the lowest in the AS group (569.23). Of these, *cysC* was the most abundant gene family, 1.7 times more abundant than *sir* and *cycH*, 3.06 times more abundant than *cycJ*. Altogether, significant differences in the total abundances of genes related to S metabolism among different regions were detected. Specifically, the LP (1587.46) and KS (1506.16) regional groups have the highest gene abundance for S metabolism, followed by QT (1496.76) and AS (1429.73), with FG (1420.98) and HC (1341.04) having the lowest abundance (Figure 5 and Supplementary Tables S12, S13).

**Table 2:** Microbial sulfur cycle processes in this study.

Name	Description	References
Assimilatory sulfate reduction	The pathway contains 11 gene families. The *cysD*, *cysN*, and *sat* gene families are involved in sulfate activation to adenosine 5′-phosphosulfate (APS), while *cysC* converts APS to phosphoadenosine 5′-phosphosulfate (PAPS). The *cysN*-*cysC* gene cluster encodes the bifunctional enzyme CysN/CysC, responsible for sulfate assimilation to PAPS. Subsequently, *cysH* reduces PAPS to sulfite, and *cysI*, *cysJ*, and *sir* reduce sulfite to sulfide	Yu et al. (2021)
Dissimilatory sulfur reduction and oxidation	The pathway contains 22 gene families. The *sat* gene family participates in the interconversion of sulfate and adenosine 5′-phosphosulfate (APS). The *aprAB* and *qmoABC* gene clusters are involved in the transformation of APS to sulfite. Furthermore, *dsr* gene families function in both dissimilatory sulfur reduction and oxidation. Specific members of these families (e.g., *dsrAB*, *dsrC*, *dsrD*, *dsrEFH*, *dsrL*, *dsrMKJOP*) are responsible for the transformation between sulfite and sulfide	Yu et al. (2021)
SOX systems	The SOX systems, comprising the 7 gene families *soxA*, *soxB*, *soxC*, *soxD*, *soxX*, *soxY*, and *soxZ*, catalyzes the oxidation of thiosulfate to sulfate in this pathway	Yu et al. (2021)
Sulfur reduction	The pathway contains 26 gene families. The *asrABC*, *fsr*, and *mccA* gene families are responsible for reducing sulfite to sulfide. The *otr* and *ttrABC* gene clusters reduce tetrathionate to thiosulfate. The *sreABC* and *psrABC* gene clusters mediate the reduction of elemental sulfur and polysulfide, respectively. Additionally, the *hydABDG*, *shyABCD*, and *sudAB* gene clusters catalyze the reduction of both elemental sulfur and polysulfide to sulfide	Yu et al. (2021)
Sulfur oxidation	The pathway contains 14 gene families. The *fccAB* and *sqr* gene families mediate sulfide oxidation. The *doxAD*, *glpE*, *sseA*, and *tsdAB* gene clusters oxidize thiosulfate, while *soeABC* and *sorAB* catalyze sulfite oxidation	Yu et al. (2021)
Sulfur disproportionation	The pathway contains 5 gene families. The *phsABC* gene cluster encodes thiosulfate reductase, which catalyzes the conversion of thiosulfate to sulfite and sulfide. The *tetH* gene mediates the disproportionation of tetrathionate into elemental sulfur, thiosulfate, and sulfate. Additionally, *sor* facilitates the transformation of elemental sulfur to sulfite and sulfide	Yu et al. (2021)

### 3.3 Effect of environmental factors on the microbial genes involved in the C, N, and S cycling

**Figure 6:**
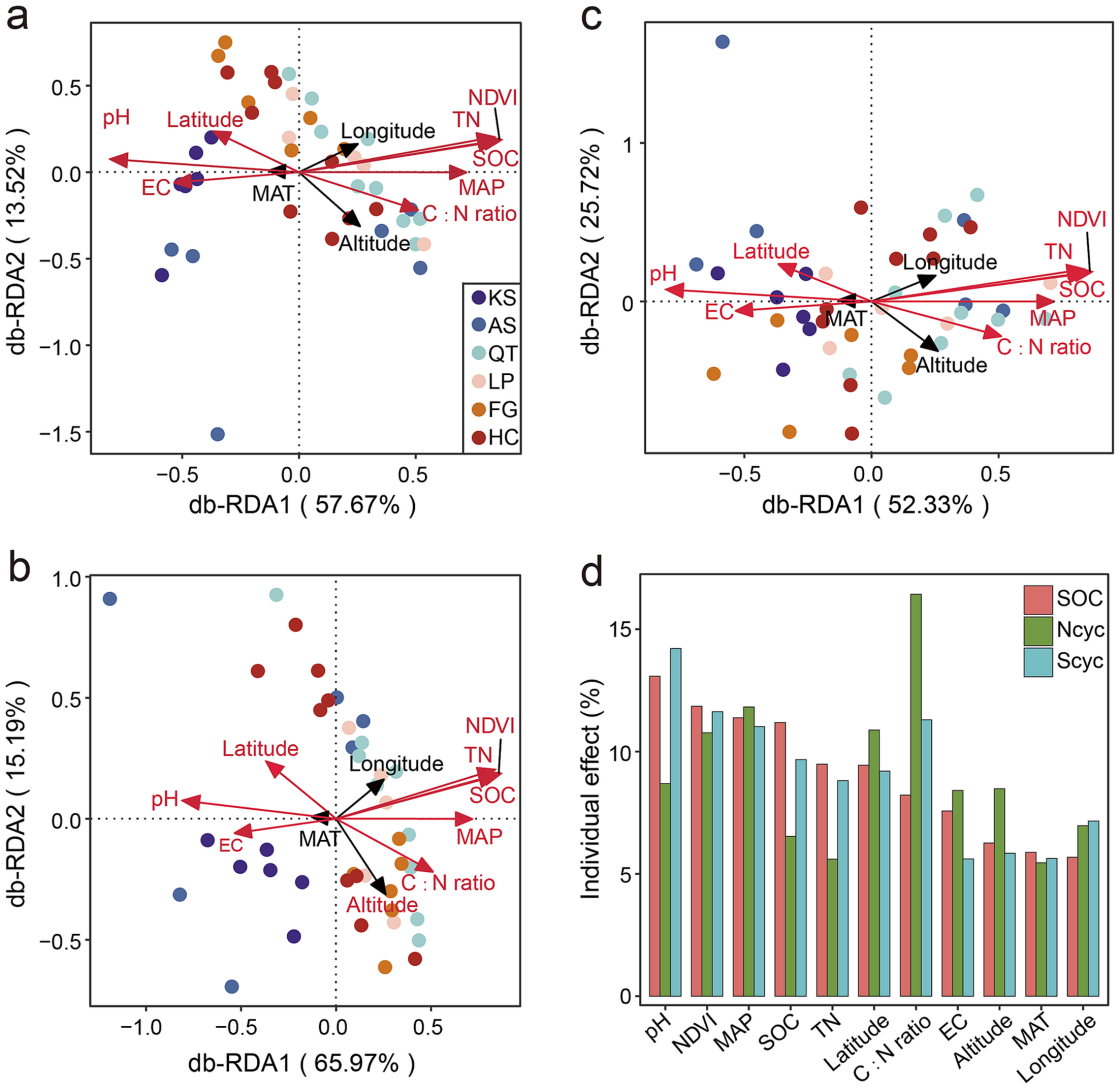
Importance of environmental factors in driving the distribution of RPKM abundance for functional trait genes. Distance-based redundancy analysis (db-RDA) illustrates the effects of environmental factors on the variations in microbial genes composition involved in the SOC **(a)**, N **(b)**, and S **(c)** cycling, respectively. **(d)** Individual impact of each environmental factor is calculated based on rdacca.hp package in C, N, and S cycling. Vectors represent environmental factors; red vectors represent environmental factors significantly correlated with the first two axes (*p* < 0.01). KS, karst area of southwest China; AS, agro-pastoral ecotone of southwest China; QT, Qinghai–Tibet Plateau; LP, Loess Plateau; FG, forest-grassland ecotone; HC, deserts of the Hexi Corridor.

To understand the relationship between the microbial abundance of the functional genes involved in C, N, and S cycling, across the six eco-regions in China, when subjected to various environmental factors, distance-based redundancy analysis (db-RDA) was used (Figure 6). The db-RDA showed that the first two axes accounted for 70.19% of the variability of the microbial genes composition involved in the C cycling, whereas db-RDA1 (the x-axis) and db-RDA2 (the y-axis) accounted for 57.67 and 13.52% of the variation, respectively (Figure 6a). For N cycling, the db-RDA showed that the first two axes accounted for 81.16% of the variability of the microbial genes composition, whereas db-RDA1 (the x-axis) and db-RDA2 (the y-axis) accounted for 65.97 and 15.19% of the variation, respectively (Figure 6b). As for the S cycling, the db-RDA showed that the first two axes accounted for 79.05% of the variability of the microbial genes composition, whereas db-RDA1 (the x-axis) and db-RDA2 (the y-axis) accounted for 53.33 and 25.72% of the variation, respectively (Figure 6c). It was interesting that latitude and seven soil properties (including pH, NDVI, MAP, SOC, TN, C:N ratio, and EC) were significantly correlated with the first two axes (*p* < 0.01) (Figure 6 and Supplementary Table S14) of the C, N, and S cycling, and these environmental factors explained 82.21, 79.13 and 81.45% (Figure 6d and Supplementary Table S14) of the variations in microbial genes composition involved in the C, N, and S cycling, respectively.

## 4 Discussion

In our study, desert (HC) and karst (KS) ecosystems harbored the most abundant distinctive KEGG pathways compared to other regions (Figure 2b and Supplementary Table S8). This phenomenon could be primarily related to the adaptation strategies of microorganisms habiting ecosystems subjected to extreme environments, namely high functional diversity and redundancy (Dong et al., 2024; Louca et al., 2018; Shu and Huang, 2022). Desert is an ecosystem considered of low-productivity, low-biomass, and polyextreme [(hyper) arid and (hyper) oligotrophic conditions with phenomena of strong ultraviolet radiation and evapotranspiration] (Coleine et al., 2024; Duan et al., 2022; Ramond et al., 2022). The karst ecosystem is also a typical oligotrophic environment due to presence of bare rocks on the surface and a thin or almost absent soil surface layer creating limiting conditions for plant growth. Moreover, karst ecosystems are mainly formed in temperate tropical regions where high temperatures accelerate soil nutrient loss and humus organic fertility is insufficient (Wang et al., 2020). Generally, microbes with contrasting life-history strategies exhibited different functional traits (Chen et al., 2021). A classical classification of microbial life history is the copiotroph-oligotroph dichotomy (Chen et al., 2021). A high nutrient (copiotrophic) strategy can be associated with greater abundance of genes related to cell division and cell cycle, while a low nutrient (oligotrophic) strategy could have a greater abundance of genes related to carbohydrate metabolism and virulence, disease and defense (Song et al., 2017). Our results are in line with a former study showing that oligotrophs may be capable of utilizing a broad range of carbon substrates (Figures 2b,c and Supplementary Table S8). For example, a series of KEGG pathways associated with carbohydrate metabolism (e.g., butanoate metabolism, amino sugar and nucleotide sugar metabolism, fructose and mannose metabolism, and galactose metabolism) were more abundant in the KS and HC regions (Figures 2b,c and Supplementary Table S8). These KEGG pathways are associated with microbes having an extraordinary ability to acquire a variety of nutrients and energy from infertile soils. Oligotrophic microorganisms had greater gene abundances associated with metabolic functions suggesting that metabolic versatility is an essential trait of oligotrophic microbial communities (Liao et al., 2023; Zheng et al., 2024). The metabolic versatility likely reflects an important adaptive strategy of oligotrophs in coping with resource scarcity (Chen et al., 2021). Moreover, soil microorganisms habiting oligotrophic ecosystems become progressively enriched with genes associated with stress-tolerant processes enabling oligotrophs to maintain genome integrity by preventing radiation-induced DNA damage in bare soils, e.g., DNA damage repair, cation transport, sporulation, and osmolyte biosynthesis (Coleine et al., 2024; Goberna et al., 2014; Malik et al., 2020). Our results showed that 5 KEGG pathways associated with *replication and repair* (Base excision repair, DNA replication, homologous recombination, mismatch repair, and nucleotide excision repair) were enriched in the HC region (Figures 2b,c and Supplementary Table S8). Thus, such harsh conditions encourage microbes in environments such as the HC or KS regions to evolve more diverse metabolic pathways to thrive in such “demanding” soil habitats.

Nitrogen (N) is considered to be a critical limiting factor in the productivity of deserts, second only to water availability, due to the extreme lack of nitrogen fertilizers and the high C:N ratio of plant litter inputs (Hu et al., 2017; Ramond et al., 2022). The accumulation of N in desert soil is mainly stored through biological N_2_ fixation (Belnap, 2002). In natural soils, biological N_2_ fixation is carried out primarily by *Klebsiella pneumonia*. Its N_2_ fixation ability can only be expressed under anoxic/anaerobic conditions with molecular nitrogen as the only carbon source (Hsu and Buckley, 2009). In our study, the HC region had the highest abundance of genes responsible for encoding enzymes of N_2_ fixation across all eco-regions assessed (Figure 4 and Supplementary Table S10). From the above it becomes clear that our results are consistent with an innovative large-scale survey in distinct terrestrial ecosystems which showed that grasslands have a strong N_2_ fixation capacity and are overwhelmingly superior to deserts (Hu et al., 2024). Nitrous oxide (N_2_O) is a potent greenhouse gas with more than 1/4 of N_2_O in atmosphere having an origin from soils, with nitrification and denitrification be the two predominant processes producing N_2_O (Tian et al., 2020). The sequential aerobic oxidation of NH_3_ to NO_3_^−^ by nitrification followed by the anoxic/anaerobic reduction of NO_3_^−^ to N_2_O via denitrification (Burgin et al., 2011; Knowles, 1982). In our study, the gene abundances for nitrification for each of the regions trialed were all much smaller than those for denitrification (Figure 4 and Supplementary Table S10). This implies that denitrification, rather than nitrification, dominates N_2_O formation from terrestrial ecosystem. However, it remains unclear which specific process between concurrent nitrification and denitrification dominates the N_2_O emission globally. Moreover, the nitrification gene abundances for denitrification from the HC region is much lower than that from the other five regions (Figure 4 and Supplementary Table S10). Based on biogeochemical theory or calculation of the Gibbs energy, oxygen reduction by nitrifiers is thermodynamically favored over NO_3_^−^reduction by denitrifiers. Previous studies showed that in drylands, scarce rainfall may rarely allow for the development of the wet anoxic soil conditions that are required for denitrification (Han et al., 2024; Krichels et al., 2023). Finally, the abundance of genes for nitrogen transport and organic N metabolism in the HC region were found also lower than those from the other five regions (Figure 4 and Supplementary Table S10). This phenomenon could be related to the oligotrophic conditions that prevail in desert ecosystems.

Sulfur (S) plays a pivotal role for numerous bio-chemical processes within the atmosphere, lithosphere, pedosphere, hydrosphere, biosphere, including the functions of all living organisms. Microorganisms drive the S cycle through oxidation, reduction and disproportionation reactions, connecting the cycles of C and N, providing energy flow and biogeochemical balance to ecosystems (Zhou et al., 2024). The S cycle involves the conversion of inorganic and organic S. In inorganic S conversion, the processes of *ASR* and *DSR*, and their key functional genes such as *sat*, *aprA*, *aprB*, *dsrA* and *dsrB* have been fully studied (Santana et al., 2021; Yu et al., 2021). *DSR* is often coupled with the oxidation of organic substrates, from volatile fatty acids (VFAs) to recalcitrant aromatic hydrocarbons (Muyzer and Stams, 2008). In our study, the abundance of genes for *ASR* in the AS is lower than that from the other five regions, but the difference was not considered significant (Figure 5 and Supplementary Table S12). Meanwhile, there was no significant difference in the abundance of the genes contributing to *DSR* across the regions. Other inorganic S forms, such as [Thio-]sulfate, tetrathionates (S_4_I), and elemental S (S_0_), require further investigation on the functional genes, pathways, and the type of microorganisms involved in biotransformation. SOX complex is an enzyme complex composed of seven core proteins *SoxABCDXYZ*, first discovered in *Paracoccus pantotrophus* and only exists in the bacterial periplasmic space (Friedrich et al., 2001). Thiosulfate is a good S source in organisms, which can produce organic sulfur through assimilation, extracellular entry or S and SO_3_^2−^ spontaneous formation of S_2_O_3_^2−^, and the most important oxidation pathway through SOX system oxidation to SO_4_^2−^ (Friedrich et al., 2001; Stoffels et al., 2012). Our results indicated that the abundance of the gene responsible for thiosulfate oxidation via the SOX complex in the HC region was lower than that in the LP region, but higher than that in the other four regions.

These functional genes are ubiquitously retrieved from a variety of habitats, and they all drive a variety of biogeochemical processes (Acinas et al., 2021; Broadbent et al., 2021; Kelly et al., 2021; Murakami et al., 2022; Nayfach et al., 2021; Sun et al., 2020; Zhang et al., 2021). In our study, the abundance of N- and S-cycling-related considerably differed between different eco-regions as opposed to the CAZyme-encoding genes’ abundance that differed geographically only slightly. Specifically, for N and S cycling for both HC and FG regions, their gene abundance was lower than those from the other four regions (Supplementary Tables S10, S12). The most important reason for this difference is the huge difference in the environmental conditions among the trialed regions. Another explanation for that discrepancy is the inherent limitation of DNA-based metagenomic technique that can only detect presence/absence of functional genes and not their expression level characteristics. Thus, complementary metatranscriptomic, metaproteomic and culture-dependent approaches could have helped uncovering microbial diversity, functional potential, and adaptations in different environments (Demin et al., 2024), on a global basis. In addition, classical quantitative polymerase chain reaction (qPCR) still remains a powerful tool for quantitative analysis of the key functional genes’ abundance involved in C, N, and S cycling (Tu et al., 2017).

## 5 Conclusion

Microorganisms play a vital key role in terrestrial ecosystems participating in biogeochemical cycling of the elements essential for life. In this study, using metagenomics and statistical tools, we identified the abundance of the functional genes from the topsoil (0–20 cm) of six typical eco-regions in China. Remarkably, the HC and KS regions harbor the most abundant distinctive KEGG pathways, including carbohydrate metabolism and replication and repair of DNA. Meanwhile, we demonstrated that the abundance of the CAZyme-encoding genes differs only slightly on a geographical basis as opposed to the abundance of N- and S-cycle related genes, which varies considerably between six eco-regions. In contrast to the excellent resilience of the HC and KS regions, the abundance of N and S cycling genes in these two regions is much lower than that from the remaining four regions. Furthermore, we linked the abundance of functional genes related to the C, N, and S cycles to multiple ecological drivers (latitude, NDVI, pH, EC, SOC, TN, C:N ratio, and MAP). Overall, these findings provide a reliable evidence base to accurately describe and characterize the functioning of soil microbiomes in terrestrial ecosystems.

## Data Availability

The datasets presented in this study can be found in online repositories. The names of the repository/repositories and accession number(s) can be found at: https://www.ncbi.nlm.nih.gov/, PRJNA765386.

## Footnotes

^1^ http://data.cma.cn/

^2^ www.illumina.com

^3^ www.majorbio.com

^4^ https://github.com/OpenGene/fastp

^5^ https://github.com/voutcn/megahit

^6^ https://metagene.nig.ac.jp/metagene/metagene.html

^7^ http://www.bioinformatics.org/cd-hit/

^8^ https://github.com/ShujiaHuang/SOAPaligner

^9^ https://github.com/bbuchfink/diamond

^10^ https://www.genome.jp/kegg/

^11^ http://hmmer.org/

^12^ http://www.cazy.org/

^13^ http://www.mothur.org/wiki/Calculators

^14^ https://CRAN.R-project.org/package=vegan

^15^ www.r-project.org

^16^ https://CRAN.r-project.org/package=pheatmap

## Glossary

MAP: Mean annual precipitation
MAT: Mean annual temperature
NDVI: Normalized difference vegetation index
SOC: Soil organic carbon
EC: Electrical conductivity
TN: Total nitrogen
RPKM: Reads per kilobase per million mapped reads
KEGG: Kyoto Encyclopedia of Genes and Genomes
CAZy: Carbohydrate-active enzymes
LDA: Linear discriminant analysis
db-RDA: Distance-based redundancy analysis
PCoA: Principal co-ordinates analysis
PPMC: Pearson’s product moment correlation coefficient
GHs: Glycoside hydrolases
GTs: Glycosyltransferases
PLs: Polysaccharide lyases
CEs: Carbohydrate esterases
AAs: Auxiliary activities
CBMs: Carbohydrate-binding modules
DNRA: Dissimilatory nitrate reduction to ammonia
ANRA: Assimilatory nitrate reduction
KS: Karst area of southwest China
AS: Agro-pastoral ecotone of southwest China
QT: Qinghai–Tibet Plateau
LP: Loess Plateau
FG: Forest-grassland ecotone
HC: Deserts of the Hexi Corridor
