# Supplementary material for: Structure and function of the topsoil microbiome in Chinese terrestrial ecosystems
Source: Front Microbiol. 2025 Aug 25;16:1595810. doi: 10.3389/fmicb.2025.1595810 (PMC12414936; doi:10.3389/fmicb.2025.1595810)
Supplement: Supplementary file 1 [file Data_Sheet_1.pdf]

Supplementary Materials

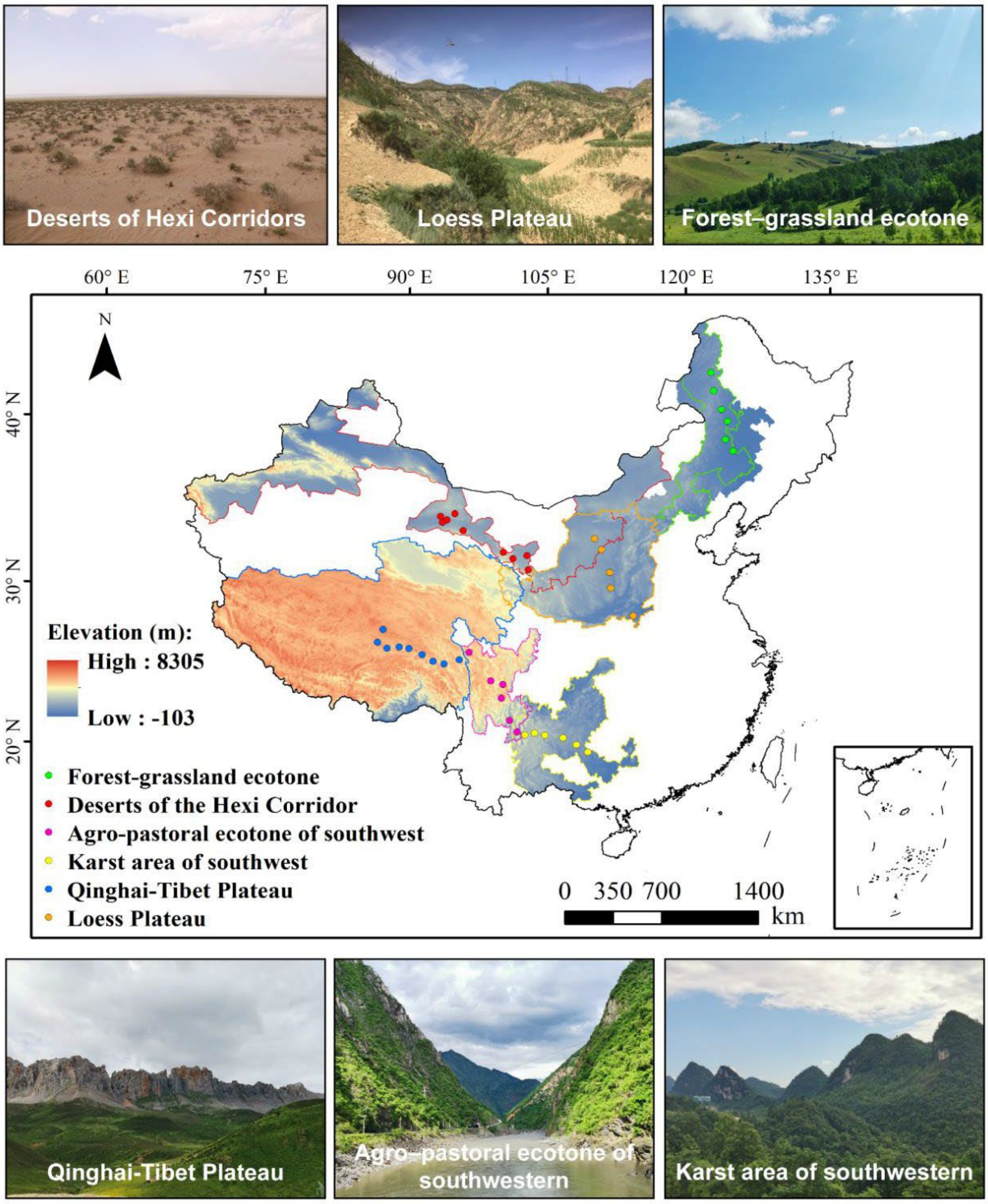

Fig. S1 Distribution of the 41 sampling sites in this study across all six eco-fragile regions of China. Regions are the karst area of southwest China (*KS*, n = 6), agro–pastoral ecotone of southwest China (*AS*, n = 6), Qinghai-Tibet Plateau (*QT*, n = 9), Loess Plateau (*LP*, n = 5), forest–grassland ecotone (*FG*, n = 6), and deserts of the Hexi Corridor (*HC*, n = 9).

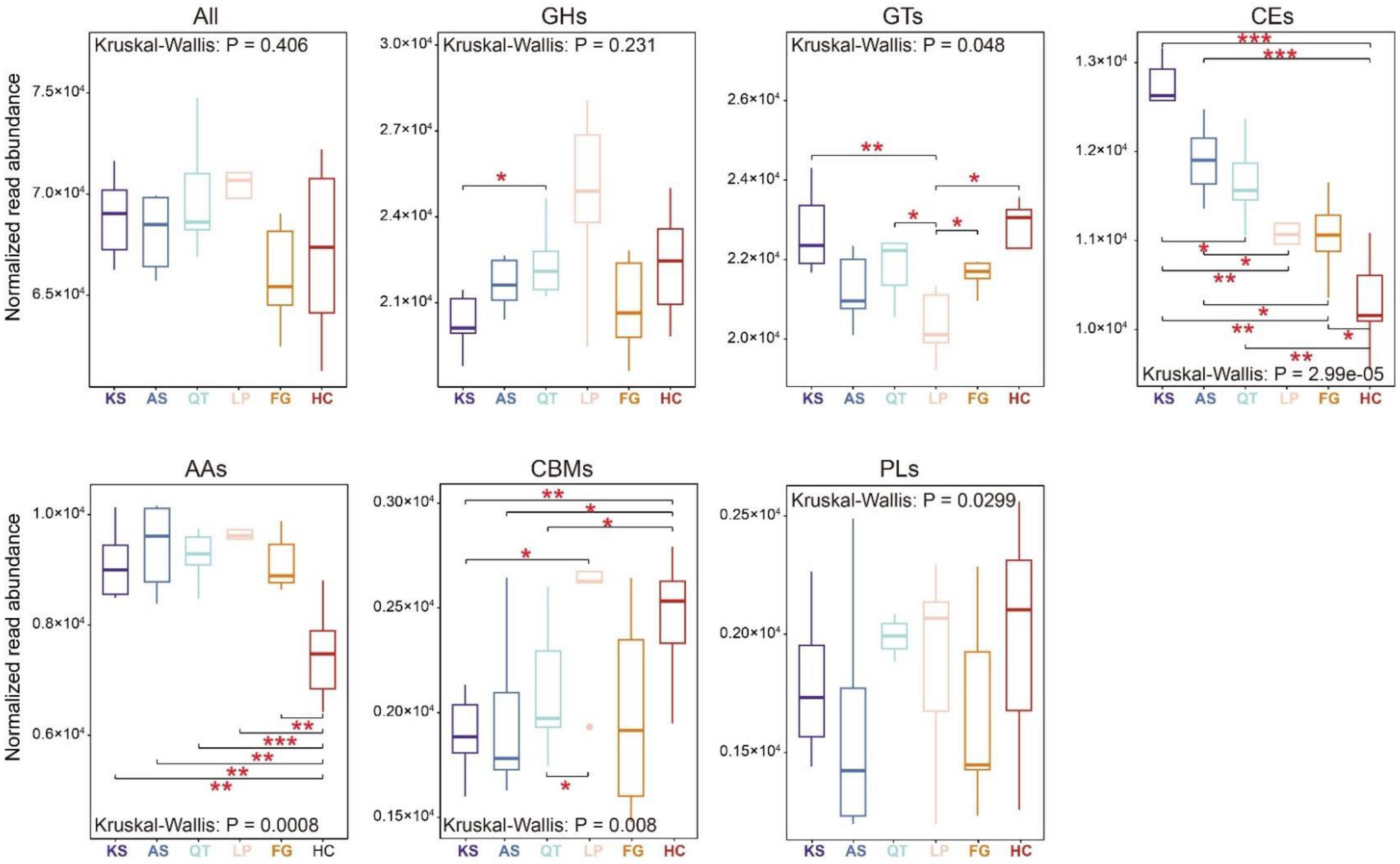

Fig. S2 Changes in CAZy enzyme genes involved in SOC (soil organic carbon) decomposition and biosynthesis across the 41 sampled soil microbiomes in six eco-regions across China. All, CAZy enzyme genes; GHs, glycoside hydrolases; GTs, glycosyl transferases; CEs, carbohydrate esterase; AAs, auxiliary activity; CBMs, carbohydrate-binding modules; PL, polysaccharide lyases. Asterisks indicate significant differences (**P*<0.05; **0.01<*P*<0.05; ***0.001<*P*<0.01) based on the analysis of variance.
